# Treatment of Landfill Leachate Using Activated Sludge Technology: A Review

**DOI:** 10.1155/2018/1039453

**Published:** 2018-09-04

**Authors:** Kai Wang, Lusheng Li, Fengxun Tan, Daoji Wu

**Affiliations:** ^1^School of Municipal and Environmental Engineering, Shandong Jianzhu University, Jinan 250101, China; ^2^Qingdao Xin Bei De Environmental Technology Co. Ltd., Qingdao 266000, China

## Abstract

Landfill leachate contains a large amount of organic matter and ammoniacal nitrogen. As such, it has become a complex and difficult issue within the water treatment industry. The activated sludge process has been found to be a good solution with low processing costs and is now therefore the core process for leachate treatment, especially for nitrogen removal. This paper describes the characteristics and treatment of leachate. Treatment of leachate using the activated sludge process includes the removal of organic matter, ammoniacal nitrogen, and total nitrogen (TN). The core method for the removal of organic matter involves anaerobic treatment supplemented with an aerobic process. Ammoniacal nitrogen is commonly removed using a conventional aerobic treatment, and advanced TN removal is achieved using endogenous denitrification or an anaerobic ammonium oxidation (ANAMMOX) process. Since biological processes are the most economical method for TN removal, a key issue is how to tap the full potential of the activated sludge process and improve TN removal from leachate. This complex issue has been identified as the focus of current scholars, as well as an important future direction for leachate research and development.

## 1. Introduction

Solid waste sanitary landfills have been the most common and most important way of dealing with garbage across the world. To take China as an example, the country's total solid waste reached 1.73 billion kilograms in 2013 and 80% of this output was processed through sanitary landfills because of the low costs associated with this method [1].

Leachate is a by-product of sanitary landfills, and, due to its large concentration of pollutants, it must be properly treated before being discharged. The total amount of leachate generated by solid waste sanitary landfills has reached 30 million tons per year. Because the waste composition is very complex, with high organic, ammoniacal nitrogen, and salt content, landfill leachate is considered to be a special wastewater [2–4]. The amount of pollutants in one ton of landfill leachate is equivalent to the amount of pollutants found in 100 tons of urban wastewater. Directly discharging leachate into the surrounding environment would cause irreversible harm, especially to groundwater systems.

Conventional sewage treatment leaves behind high concentrations of ammoniacal nitrogen in landfill leachate which can cause the eutrophication of water bodies. Although biochemical treatments are used to reduce the ammoniacal nitrogen concentration to agreed levels, nitrite concentration in leachate can remain high. Nitrite is a recognized carcinogen; if attention is only given to the control of ammoniacal nitrogen and TN is neglected, the detrimental effects of leachate on the environment could be substantial. Implementing more stringent emission standards for TN in leachate is therefore imperative for countries that want to protect their local environment. In 2008, China revised and implemented new leachate emission standards (GB16889-2008). The new standard increased regulations on the discharge of TN, total phosphorus, and six heavy metal indicators. The requirements of these regulations, especially in relation to TN emissions, are both a challenge and an opportunity for leachate treatment. The challenge is that they are more stringent, increasing the difficulty associated with leachate disposal. However, on the other hand, the new standards will accelerate the development and promotion of new technologies.

## 2. The Characteristics of Landfill Leachate

Landfill leachate is a foul-smelling black or brown liquid. It contains large amounts of organic and inorganic material, including a number of refractory organics such as aromatic compounds and humus; inorganic salts such as ammoniacal nitrogen, carbonate, and sulfate; and metal ions such as chromium, lead, and copper [5, 6]. Because of the complex composition of the waste, a characteristic of leachate water quality is that it contains high levels of contaminants and, often, biological toxicity.

As a result, chemical oxygen demand (COD) in leachate is typically above 20000 mg/L. Besides toxic aromatic compounds, leachate is also rich in organic macromolecules such as humus and humic acid. Ammoniacal nitrogen concentration above 2000 mg/L is often achieved. This toxic organic matter and these high ammoniacal nitrogen levels cause difficulty during processing, especially for biological treatments. Even in the absence of toxicity, organisms cannot achieve effective microbial degradation because of the large molecular weight and insufficient chemical stability. Therefore, an activated sludge process cannot achieve effective reduction of COD and an advanced treatment process must therefore be developed.

Another feature of leachate is the variance in the quality and quantity of wastewater from different landfills; location has a significant impact. Relatively speaking, the concentration of leachate pollutants in the United States and Europe is much lower than in Asian countries. For example, ammoniacal nitrogen in leachate from European and American countries is generally below 1000 mg/L, while it is generally above 1000 mg/L in Asian countries [7–12]. These differences may relate to different cultures and behaviors in the various regions. In addition, leachate quality can differ in the same place at different times and can be divided into early (less than five years old), medium-term (5–10 years old), and old landfill leachate (more than 10 years old) [13]. Leachate characteristics identified at different times are presented in Table 1 where the values in parentheses represent typical levels.

As shown in Table 1, the features of early leachate are high organic content, strong biodegradability, and relatively low ammoniacal nitrogen concentration. The features of old leachate are high ammoniacal nitrogen content, little biodegradability, and poor COD/NH_4_^+^-N (or carbon-to-nitrogen ratio). The quality of medium-term leachate water is somewhere between that of the early and old leachates [14, 15]. Meanwhile, the quantity of leachate in the same area is larger in the rainy season and contains higher organic content. The amount of leachate is much reduced in the dry season and it contains high ammoniacal nitrogen concentrations. The third characteristic of leachate water quality is nutritional imbalance; organic matter, ammoniacal nitrogen, and heavy metal concentrations are very high but phosphorus content is very low. Low phosphorus content and concentrated heavy metals increase the difficulty of developing an effective biological treatment for leachate.

## 3. Treatment of Landfill Leachate and Difficulty

### 3.1. Treating Landfill Leachate

Since leachate contains large amounts of organic matter and ammoniacal nitrogen, general disposal methods have included physical and chemical pretreatment followed by aerobic and anaerobic biochemical processes, concluding with further physical and chemical methods for final in-depth treatment.

The main functions of pretreatment are to remove suspended solids, degrade some of the organic matter and ammoniacal nitrogen, reduce toxicity, and improve the overall biodegradability of the leachate. This is achieved through coagulating and stripping the ammoniacal nitrogen from the leachate. The function of the subsequent biochemical stage is to remove the biodegradable organic matter and ammoniacal nitrogen. The core technologies in these biochemical processes are, for example, the upflow anaerobic sludge blanket (UASB), membrane bioreactors (MBR), the anoxic-oxic (A/O) process, and sequencing batch reactors (SBR). The later deep treatment of leachate further removes organic matter and TN and can include Fenton oxidation, electrochemical processes, activated carbon adsorption, and membrane treatment processes.

This largely biochemical disposal method results in most biodegradable organic compounds and ammoniacal nitrogen being removed, along with a portion of TN. However, the leachate water still contains a large amount of refractory organic compounds and some TN. In order to achieve current discharge standards, double membranes using nanofiltration and reverse osmosis are employed as safeguards.

### 3.2. Difficulties in Treating Landfill Leachate

At present, the main difficulties in leachate treatment are as follows:

1. Leachate has high organic content and a large amount of toxic and organic molecules. Discharge standards cannot be achieved using a single biochemical or physicochemical process; a combination of physicochemical and biochemical processing is required. Choosing a reasonable, economical, and efficient combined process is the first challenge
2. Ammoniacal nitrogen levels are high, and identifying an effective and complete nitrogen removal process for leachate is difficult. Traditional biological treatment processes can effectively remove ammoniacal nitrogen, but it is not ideal for TN removal. Improving the efficiency of TN removal by biological treatment process is the second key difficulty
3. The significant changes in water quality and quantity increase the difficulty of identifying a stable standard discharge method. In different seasons, leachate water quality and quantity can be very different which challenges both the selection and the operation of a suitable treatment process. Identifying a suitable combination of available technologies and how to use them to ensure a stable operation are the third challenge in leachate treatment
4. The treatment process is complex and the costs are very high. In order to achieve discharge standards, leachate treatment plants often use nanofiltration and reverse osmosis which makes treatment costs high. Reducing costs in leachate treatment is the fourth main difficulty

To summarize, it is necessary to identify the appropriate combination of biochemical and physicochemical treatments to dispose of landfill leachate. In addition, maximizing the potential of the biochemical treatment process, improving the TN removal rate, and reducing the total costs are the main challenges associated with developing leachate treatment processes.

## 4. The Removal of Organic Matter by Activated Sludge

Leachate contains a considerable amount of both biodegradable and nonbiodegradable organic matters. Activated sludge processes can effectively remove biodegradable organic material by completely converting it to carbon dioxide and water. The process can involve anaerobically or aerobically activated sludge. The advantages of an anaerobic process are that it has low energy consumption and can produce energy itself. The disadvantages are that effluent COD is high and retains some biodegradable organic matter. Alternatively, the advantages of an aerobic process are high biodegradation and organic removal rate, as well as good water quality. A disadvantage is high energy consumption throughout the process.

### 4.1. Anaerobic Activated Sludge Process

The anaerobic activated sludge process for treating landfill leachate can include upflow anaerobic sludge blanket (UASB), anaerobic membrane bioreactor (MBR), and expanded granular sludge blanket reactor (EGSB). The efficiency of organic removal by anaerobic activated sludge processes is shown in Table 2.

The UASB process results in high removal efficiency and large volumetric loading. This method is therefore often used to treat leachate with high organic content. Agdag and Sponza report on the use of a UASB to dispose of landfill leachate [16]. The hydraulic retention time (HRT) was 1.25 days and the COD removal rate was 80%. Peng et al. combined two processes to process leachate, using a UASB alongside the anoxic-oxic (A/O) process, which resulted in COD of 8550 mg/L–12500 mg/L [17]. The total volumetric loading of the UASB reached 21 kg COD/m^3^·d and COD was reduced by more than 80%.

Callia et al. also used a UASB to dispose of leachate observing COD levels of 12350–47800 mg/L [18]. The volumetric loading of their UASB reached 23.5 kg COD/m^3^·d and the COD removal rate was 80%. Bohdziewicz and Kwarciak used UASB to dispose of leachate, which saw the COD at 3500 mg/L–4200 mg/L [19]. The influent included 20% wastewater. The final HRT was two days and the volumetric loading was 2 kg COD/m^3^·d. The final removal of organic waste was above 76%. Compared to the results of Callia et al., the low volumetric loading of Bohdziewicz's UASB was due to the lower levels of organic matter in the influent.

Anaerobic MBR contain a high concentration of sludge and the effluent quality from this process is good. Bohdziewicz et al. used an anaerobic MBR to process influent that comprised 20% leachate and 80% wastewater [20]. COD was observed at 2800–5000 mg/L and COD was reduced by up to 95%. The HRT and organic loading rate of the MBR were two days and 2.5 kg COD/m^3^·d, respectively. Xie et al. also used an anaerobic MBR to dispose of leachate, observing a COD level at 13000 mg/L and an ammoniacal nitrogen level at 3000 mg/L [21]. The average COD removal rate was 62% while the volumetric loading was 4.87 kg COD/m^3^·d. Furthermore, the results demonstrated that *Alkaliphilus*, *Petrimonas*, *Fastidiosipila*, and vadin BC27 were the abundant fermentation bacteria found in the bacterial communities.

The EGSB, a third-generation anaerobic reactor, has the characteristic of high volumetric loading. Liu et al. studied the effects of ammoniacal nitrogen concentration on organic matter removal efficiency when using an EGSB to dispose of landfill leachate [22]. The results showed that the influent's average COD was around 33000 mg/L and the EGSB's maximum volumetric loading was 64 kg COD/m^3^·d. The COD removal rate was 85%–90%. When the concentration of ammoniacal nitrogen was under 1500 mg/L, the removal rate of COD was slightly affected.

### 4.2. Aerobic Activated Sludge Process

The aerobic activated sludge process for treating leachate includes sequencing batch reactors (SBR), aerobic membrane bioreactors (MBR), the A/O process, and biofilm reactors. The efficiency of aerobic activated sludge processes in removing organic matter from landfill leachate is shown in Table 3.

SBR are the predominant technology used in landfill leachate treatment because of their simple structure and large capacity. Klimiuk et al. used an SBR to process leachate which saw COD at 1348 mg/L [23]. With an HRT of 12 hours, COD was reduced by 80%–85% and a 5-day biochemical oxygen demand (BOD_5_) was reduced by more than 99%. By increasing the filler in an SBR, its handling capacity can also be increased. Lim et al. used rice husks as filler in an SBR, thereby observing COD at 1040–4870 mg/L and a COD removal rate of over 70% [24].

MBR are often used to treat leachate because of their high sludge concentration and good effluent water quality. Zolfaghari et al. used an MBR that saw COD at 2200 mg/L and COD removal rate was stabilized at 90% [25]. A high concentration of activated sludge and rich microbial populations provide a good foundation for the reduction of COD. Sanguanpaka et al. studied the treatment efficiency of an MBR using water with different pH levels [26]. The average COD of the influent leachate was 5445 mg/L when initial pH levels ranged between 5.66 and 8.79. Changes to the COD removal rate were small with levels maintained at 98.1%–99.25%.

The A/O process is often used to treat leachate because of its strong nitrogen removal; the rate of COD reduction is also very good when A/O is used to process landfill leachate. Peng et al. used a UASB and the A/O process (UASB + A/O) whereby effluent from the UASB enters an A/O system to further reduce COD [17]. The A/O influent's COD was 2000–3000 mg/L and the effluent's COD was around 1500 mg/L; COD was therefore reduced by more than 40%. The combined UASB + A/O system delivered COD and BOD_5_ removal rate of 80%–92% and 99%, respectively.

At present, the aerobic activated sludge process is used to remove ammonium from leachate. However, the efficacy of COD reduction is also very important. Once aerobic treatment of leachate is complete, biological organisms can be almost completely removed. Consequently, threats to the environment caused by landfill leachate are significantly reduced.

### 4.3. Summary of Activated Sludge Processes

The removal of organic matter by an activated sludge process is considered the most effective and economical way of achieving the desired outcome. The low energy consumption of the anaerobic process combined with the efficiency of the aerobic method could greatly reduce the environmental harm caused by leachate. However, due to the complex composition of leachate, large amounts of organic matter remain in the final effluent discharged after these biological treatments and this makes it difficult to reach regulatory standards. Further development and more effective disposal methods are yet required.

## 5. The Removal of Ammoniacal Nitrogen by Activated Sludge

Ammoniacal nitrogen found in leachate typically exceeds 1000 mg/L, although some leachates contain even higher levels of up to 3000 mg/L. Significant discharge of ammoniacal nitrogen directly into the surroundings can cause great harm to the environment and especially local groundwater systems. Many developed countries have devised strict emission standards in regard to landfill leachate. In 1997, China implemented one such set of regulations (GB16889-1997). The standards stipulate specific permitted discharge levels of suspended solids, BOD_5_, COD, ammoniacal nitrogen, and *E. coli*. As a result, the cost-effective removal of ammoniacal nitrogen from landfill leachate has been a significant challenge for the water treatment industry. Accordingly, activated sludge processes have been key methods in ammoniacal nitrogen disposal because of the low associated costs and low secondary pollution.

### 5.1. Ammoniacal Nitrogen Removal by SBR

SBR are the preferred process used for landfill leachate ammoniacal nitrogen disposal. Lo used SBR to dispose of landfill leachate and the ammonia nitrogen removal rate was 99% [27]. Similarly, Spagni and Marsili-Libelli used an SBR to process leachate and observed an average COD of 2055 mg/L [28]. The average level of ammoniacal nitrogen was 1200 mg/L, and the shortcut nitrification and ammoniacal nitrogen removal rates reached 98% and 99%, respectively. Because of a significant imbalance in the carbon-to-nitrogen (C/N) ratio in the leachate, the test used an additional carbon source to achieve denitrification. The TN removal efficiency was more than 95%.

Aziz et al. employed two different SBR to process landfill leachate—one with powdered activated carbon (PAC) and one without [29]. The average COD and average ammoniacal nitrogen in the leachate were 1396 mg/L and 579 mg/L, respectively. Without PAC, the ammoniacal nitrogen removal rate of the SBR was 85.5%. This increased to 89.4% with the addition of PAC; the energy-saving effect is evident.

Sun et al. investigated an SBR's capacity for removing ammoniacal nitrogen at low temperatures [30]. In their study, the leachate's average COD and ammoniacal nitrogen levels were 665 mg/L and 155 mg/L, respectively. The results showed that even at low temperatures of between 13 and 17.6°C, the system achieved rapid shortcut nitrification and ammoniacal nitrogen was removed at a rate of more than 99%. The TN removal rate reached 90% with the addition of a carbon source. Sun et al. studied the effect of using a combination of a UASB and an SBR to treat leachate [31]. The influent COD and ammoniacal nitrogen were 1237–13500 mg/L and 738–2400 mg/L, respectively. The results showed that the system's ammoniacal nitrogen removal rate reached 99.5% and, after adding external carbon to the SBR, the TN removal rate exceeded 99.1%.

Granular sludge sequencing batch reactors (GSBR) provide a new process with high rates of nitrogen removal. Ren et al. report on the use of a GSBR in leachate treatment, resulting in ammoniacal nitrogen levels of 498 mg/L at a removal rate of more than 99% [32]. The microenvironment of the granular sludge was found to achieve good simultaneous nitrification and denitrification, with the GSBR's TN removal rate reaching 50%–60%.

### 5.2. Ammoniacal Nitrogen Removal by MBR

Canziani et al. used an MBR to dispose of leachate which averaged COD and ammoniacal nitrogen levels at 6361 mg/L and 1497 mg/L, respectively [33]. Ammoniacal nitrogen was removed at a rate of 95% and a stable shortcut nitrification rate of 90% was achieved. Zolfaghari et al. used a sequencing batch MBR in their study [34]. COD in the leachate was 1550 mg/L–2122 mg/L and ammoniacal nitrogen was 288 mg/L–434 mg/L. The results showed that the COD and ammoniacal nitrogen removal rates in summer were 63.4% and 98.2%, respectively. The COD and ammoniacal nitrogen removal rates in the winter were 53.2% and 99.2%, respectively. Zhang et al. used a combination of an MBR with Fenton oxidation and reverse osmosis to process leachate [35]. COD of the MBR influent was around 1500 mg/L, and the ammoniacal nitrogen level was between 600 mg/L and 700 mg/L. The COD removal rate of MBR was more than 95% and the ammoniacal nitrogen removal rate was more than 80%.

Additionally, Remmas et al. studied MBR leachate treatment, observing an average COD level of 1600 mg/L and an average ammoniacal nitrogen level at 600 mg/L [36]. In order to ensure the success of the tests, the researchers used diluted leachate at the beginning of the study. When the process was considered stable, the proportion of leachate was gradually increased until the influent was composed entirely of leachate. The rates of reduction in COD and ammoniacal nitrogen were more than 50% and 95%, respectively, when ammoniacal nitrogen levels were below 600 mg/L. When ammoniacal nitrogen in the influent was above 800 mg/L, the removal rate clearly declined, indicating that high ammoniacal nitrogen levels impact the stability of the system. After denitrification through the addition of carbon, the TN removal rate was 80%–90%.

### 5.3. Other Processes to Remove Ammoniacal Nitrogen

There are many activated sludge methods used to dispose of leachate in addition to SBR and MBR. These include the conventional continuous flow and A/O processes, the use of rotating biological contactors (RBC), and sequencing batch biofilter granular reactors (SBBGR), as well as combinations of activated sludge processes.

The continuous flow process has a simple construction and a high rate of ammoniacal nitrogen removal. Yusof et al. employed a continuous flow process to process leachate, and they report average COD and ammoniacal nitrogen levels of 2897 mg/L and 1452 mg/L, respectively [37]. The final ammoniacal nitrogen volumetric loading was 3 kg N-NH_4_^+^/m^3^·d and the removal rate was 99%. Effluent nitrate was maintained at around 1200 mg/L. Elsewhere, Halim et al. used a fixed-bed column process achieving average COD and ammoniacal nitrogen levels of 2580 mg/L and 1030 mg/L, respectively [38]. The reduction in COD and ammoniacal nitrogen reached rates of 92.6% and 86.4%, respectively. After system regeneration, the rates of reduction in COD and ammoniacal nitrogen increased to 93.7% and 90.0%, respectively.

Because the A/O process has both nitrification and denitrification applications, it can remove not only ammoniacal nitrogen but also TN by using a reflux nitrification liquid. As previously outlined, Peng et al. used a UASB + AO process to treat landfill leachate [17]. The ammoniacal nitrogen level after A/O process was 1100–2000 mg/L, and the ammoniacal nitrogen removal rate was 99%. The maximum ammonia nitrogen removal volumetric loading was 0.68 kg N-NH_4_^+^/m^3^·d. Through the denitrification process of UASB + AO, the TN removal rate was 91–93%. Wu et al. also used UASB + AO to dispose of leachate which averaged COD and ammoniacal nitrogen levels at 9500 mg/L and 2000 mg/L, respectively, and the rate of ammoniacal nitrogen removal was over 97% [39]. By using a denitrification process in the anoxic zone of the A/O process, a TN removal rate of 80–85% was achieved.

Chen et al. modify the A/O process to process the leachate in their study; an anoxic tank was added after the aerobic tank for denitrification [40]. The average COD and ammoniacal nitrogen levels of the leachate were 3144 mg/L and 1425 mg/L, respectively. The ammoniacal nitrogen and TN removal rates of this system were 95% and 66.4%, respectively. The shortcut nitrification rate was maintained at 90%.

RBC is easily managed and has low consumption. Kulikowska et al. used two RBC processes to treat leachate which averaged the ammoniacal nitrogen concentration level at 834 mg/L [41]. The single-stage RBC could achieve good nitrification when ammoniacal nitrogen volumetric loading was at 1.92 g N-NH_4_^+^/m^2^d and the rate of ammoniacal nitrogen removal exceeded 99%. When the ammoniacal nitrogen volumetric loading was at 3.6 g N-NH_4_^+^/m^2^d, two RBC processes were required to achieve complete nitrification, and when the ammoniacal nitrogen volumetric loading was at 4.79 g N-NH_4_^+^/m^2^d and 6.63 g N-NH_4_^+^/m^2^d, the removal rate decreased to 74.4% and 71.6%, respectively [41].

The SBBGR is a new type of activated sludge process which is characterized by a high concentration of sludge and effective leachate treatment. Iaconi et al. employed an SBBGR to dispose of leachate and observed COD and ammoniacal nitrogen levels at 2200–3200 mg/L and 1500–2000 mg/L, respectively [42]. The ammoniacal nitrogen removal rate of the reactor reached over 99%, and the TN removal rate reached more than 99% by the addition of an external carbon source.

Because of the large amount of contaminants in leachate and their complex components, some studies use a combination of processes to ensure the effectiveness of the treatment. For example, Liu et al. studied a two-stage A/O and MBR process to treat leachate; the MBR replaced the secondary sedimentation tank in the standard A/O process [43]. This ensures not only that the sludge concentration of the system is maintained but also that the removal of COD and ammoniacal nitrogen is optimized. In this study, COD and ammoniacal nitrogen were at 4000–20000 mg/L and 1450–2100 mg/L, respectively. The ammoniacal nitrogen and TN removal rates reached 99.04% and 74.87%, respectively. High-throughput sequencing analysis indicated that Proteobacteria (44.57–50.36%), Bacteroidetes (22.09–27.25%), Planctomycetes (6.94–8.47%), Firmicutes (3.31–4.53%), and Chloroflexi (3.13–4.80%) were the dominating phyla in the system's bacterial community.

### 5.4. Summary of Ammoniacal Nitrogen Removal by Different Activated Sludge Processes

Since ammoniacal nitrogen has strong chemical stability, it is very difficult to remove it through standard physical or chemical methods. Activated sludge processes are therefore the main technologies used for the removal of ammoniacal nitrogen today. Whichever process is used, most ammoniacal nitrogen found in leachate can be effectively removed through acclimated nitrification of or nitrifying bacteria. Because high levels of ammonia nitrogen have high toxicity, in an actual application, the influent ammonia nitrogen load is very important to understand and control. A high ammonia nitrogen load may poison microbes and reduce the removal rate of the system.

## 6. The Removal of TN by Activated Sludge

Conventional activated sludge processes might achieve the ammoniacal nitrogen emission standards for landfill leachate. However, leachate organic matter is depleted during the nitrification process which poses significant challenges for traditional denitrification processes. In order to solve the problem of TN removal, a number of researchers identified that adding a further carbon source could initiate advanced denitrification. However, the cost of this approach was considered too high and not applicable in engineering scenarios. In order to reduce processing costs for TN removal, researchers have used more advanced treatment processes in recent years such as endogenous denitrification (ED) and the anaerobic ammonium oxidation process (ANAMMOX). These processes can not only meet the requirements of leachate TN removal but also have low associated costs. This is of great significance to meet the needs of the industry and to promote further development of landfill leachate treatments.

### 6.1. TN Removal by Endogenous Denitrification

Denitrifying bacteria are able to maintain a carbon source during leachate treatment. When sewage has no external source of carbon on which it might draw, this kind of bacteria uses internal carbon sources from within itself for denitrification. If this characteristic could be successfully enhanced, advanced denitrification could be achieved for landfill leachate without the addition of an external carbon source.

Zhu et al. used an aerobic sequencing batch reactor (ASBR) and an SBR to treat early landfill leachate [44]. Influent COD and ammoniacal nitrogen levels were at 8528 mg/L and 1154 mg/L, respectively. The primary role of the ASBR was to regulate the leachate's C/N ratio. The SBR influent C/N ratio was around four to one. After the first filling, the SBR was stirred and an aeration nitrification process was generated. After the last aeration, agitation continued until the system had completely removed the TN. The main purpose of premixing the raw water was to maintain a carbon source for denitrification, and the last agitation was performed to utilize the internal carbon source. The system achieved COD and TN removal rates of 89.61–96.73% and 97.03–98.87%, respectively, without any external source of carbon required.

Wang et al. also used an ASBR and SBR system to treat early leachate with COD and ammoniacal nitrogen levels of 6000 mg/L and 1100 mg/L, respectively [45]. Similar to the study of Zhu et al. above, the primary role of the ASBR here was to regulate the leachate's C/N ratio. However, in contrast to that study of Zhu et al., the SBR in this study was operated in an influent-stirring-aeration-stirring-sedimentation-draining process. After stirring the leachate, denitrifying bacteria would absorb the carbon and convert it into an internal form such as PHB. When nitrification was complete, the denitrifying bacteria used this stored carbon to realize ED. The system's COD and TN removal rates were 90% and 95%, respectively.

In conclusion, advanced nitrogen removal can be achieved through endogenous denitrification. The disadvantage of this process is that it can only treat early and medium-term leachates with a C/N ratio that is greater than four. If the C/N ratio is below four, the technology cannot be used.

### 6.2. TN Removal by Anaerobic Ammonium Oxidation

ANAMMOX is an advanced autotrophic denitrification process. Its biggest advantages are that it requires no carbon source and that TN removal efficiency is high. The main difficulty related to this process is the source of nitrite.

ANAMMOX currently used for treating landfill leachate has two major categories: one-stage ANAMMOX and two-stage ANAMMOX. One-stage ANAMMOX achieves autotrophic denitrification in one reactor; having a small number of reactors is an advantage, but control is difficult. Two-stage ANAMMOX, which involves short nitrification and anaerobic ammonium oxidation, is performed in two reactors with two functions. The first reactor realizes semishortcut nitrification and the second reactor enables ANAMMOX itself; shortcut nitrification occurs in the first reactor and the effluent is mixed with raw water to become the influent of the ANAMMOX reactor. The advantage of two-stage ANAMMOX is that bacteria are highly enriched enabling higher nitrogen removal efficiency. Its complexity is its disadvantage.

#### 6.2.1. Semishortcut Nitrification by Activated Sludge

SBR are especially conducive to realizing semishortcut nitrification in landfill leachate. Ganigué et al. used an SBR to process leachate and observed ammoniacal nitrogen levels of 1623 mg/L [46]. By controlling the alkalinity of the leachate, the ammonia nitrogen volumetric loading was kept at 1–1.5 kg N-NH_4_^+^/m^3^·d. The effluent supported stable semishortcut nitrification, and the ratio of nitrite nitrogen and ammoniacal nitrogen was 6 : 4. Meanwhile, the nitrate concentration was very low due to the high water temperatures and dissolved oxygen, which was less than 5% of water TN. In 2012, Ganigué's research group published a report in *Bioresource Technology* about the semishortcut nitrification of leachate [14]. At 6000 mg/L, the ammoniacal nitrogen level of the leachate in this study was higher than that in previous research. The results showed that, at 25°C and 35°C, stable semishortcut nitrification was enabled by controlling the ratio of alkalinity to ammoniacal nitrogen. The final effluent's ammoniacal nitrogen to nitrite ratio could be controlled at 4 : 3 which provided a good basis for ANAMMOX. Li et al. also used an SBR to treat leachate [47]. In contrast to Ganigué et al., Li et al. identified that semishortcut nitrification was mainly controlled by the amount of aeration in the SBR and the pH level of the effluent. The average ammoniacal nitrogen of the leachate in Li et al.'s study was 1748 mg/L. When aeration at 19.6 ± 171 m^3^·air/m^3^·h was applied, the volumetric loading of ammoniacal nitrogen reached 0.71 ± 0.14 kg N-NH_4_^+^/m^3^·d. To achieve stable semishortcut nitrification, the effluent pH range was adjusted according to the different ammoniacal nitrogen volumetric loads. This was generally between 8.18 and 8.39.

Thus, there are two ways to realize semishortcut nitrification–adjust the alkalinity of the leachate or the pH level of the effluent. Due to significant differences in water quality of leachate, it is difficult to maintain stable semishortcut nitrification by only controlling the pH and alkalinity of the leachate. How to realize stable semishortcut nitrification requires further exploration and innovation.

#### 6.2.2. One-Stage and Two-Stage ANAMMOX

Wen et al. used a one-stage sequencing batch biofilm reactor (SBBR) ANAMMOX process to process leachate [48]. The study investigated the TN removal capacity of the SBBR under different dissolved oxygen conditions. The results showed that when the dissolved oxygen was controlled at 2.7 mg/L, the TN removal rate was at its highest and stabilized at 90%. Thus, dissolved oxygen is very important in one-stage ANAMMOX.

To inhibit the effects of dissolved oxygen on ANAMMOX bacteria, Xu et al. used an intermittent aeration one-stage SBR to treat old leachate [49]. Short nitrification occurred when the SBR was aerated and ANAMMOX occurred when the SBR was stirred. The dissolved oxygen was controlled at 1.0–1.5 mg/L during the aeration process. Ultimately, the TN removal efficiency of the SBR exceeded 90%. The TN effluent mainly included nitrate. The activities of aerobic ammonium oxidization, anaerobic ammonium oxidization, and denitrification reached 2.83 kg NH_4_^+^-N/kg_dw_/day, 0.65 kg NH_4_^+^-N/kg_dw_/day, and 0.11 kg NO_3_^−^-N/kg_dw_/day, respectively.

Similarly, Zhang et al. used a one-stage intermittent aeration SBR process to treat leachate in their study and observed COD and ammoniacal nitrogen levels at 1900 ± 200 mg/L and 1950 ± 250 mg/L, respectively [50]. An ammonium conversion efficiency of 99.3 ± 0.3% and a TN removal efficiency of 99 ± 0.1% were subsequently obtained. Based on the nitrogen balance, the nitrogen removal contribution was 77.1% for ANAMMOX and 15.6% for denitrification. Thus, intermittent aeration could resolve disturbances from dissolved oxygen on the ANAMMOX bacteria but manipulation is very complex.

The two-stage ANAMMOX is more complicated than the one-stage version but removal efficiency is much higher. Miao et al. used three SBR in the treatment of leachate which saw COD and ammoniacal nitrogen levels at 2200 ± 200 mg/L and 2000 ± 200 mg/L, respectively [51]. The system included a carbon removal SBR, a shortcut nitrification SBR, and an ANAMMOX SBR. The carbon removal SBR uses simple aeration to remove organic matter and therefore ensure anaerobic ammonium oxidation activity which would be otherwise inhibited. The function of the shortcut nitrification SBR is to provide nitrite for the ANAMMOX SBR via a shortcut nitrification process, and the ANAMMOX SBR completes the process with final denitrification by ANAMMOX. The TN removal of the system was 90% and the ammoniacal nitrogen volumetric loading and ammoniacal nitrogen removal volumetric loading were 0.81 kg N-NH_4_^+^/m^3^·d and 0.76 kg N-NH_4_^+^/m^3^·d, respectively. In 2016, Miao used a two-stage SBR and SBBR process which measured COD and ammoniacal nitrogen levels at 3000 ± 100 mg/L [52]. The SBR served to remove organic matter and realize shortcut nitrification, and the function of the SBBR was achieved using ANAMMOX, changing the traditional mode of operation. It took five hours to fill the system; the aim of prolonging the filling time was to avoid the inhibition of nitrite for the ANAMMOX bacterium. By changing the mode of operation, the TN removal rate exceeded 95% and the effluent TN was below 20 mg/L. Adding fillers significantly improved the efficiency of the nitrogen removal in the system.

Li et al. used an SBR plus UASB process, achieving stable semishortcut nitrification in the SBR by adjusting the pH of the effluent water [53]. ANAMMOX stability was then achieved in the UASB, and, ultimately, the ammoniacal nitrogen volumetric loading was 1 kg N-NH_4_^+^/m^3^·d and the TN removal rate was 85 ± 1%. The ammonia-oxidizing bacteria (AOB) in the partial nitration SBR was mainly affiliated with *Nitrosomonas* sp. IWT514 and *Nitrosomonas eutropha*. The anaerobic AOB in the ANAMMOX reactor were mainly affiliated with *Kuenenia stuttgartiensis* [53].

Wang et al. used an A/O + UASB system to process leachate which saw COD and ammoniacal nitrogen levels of 2305 mg/L and 1240 mg/L [54]. The function of the A/O process was anoxic denitrification and shortcut nitrification. The A/O effluent entered an intermediate tank and then entered the UASB along with raw water. The COD and TN removal rates were 62% and 94%, respectively. In quantitative PCR reactions, the proportions occupied by AOB, nitrite-oxidizing bacteria, and ANAMMOX in the A/O were 11.39%, 1.76%, and 0.05%, respectively, and the proportions in the UASB were 0.35%, 4.01%, and 7.78%, respectively.

Wu et al. used a more complex UASB + AO + UASB system and observed COD and ammoniacal nitrogen levels of 2500–3000 mg/L and 1900-2000 mg/L, respectively [55]. The function of the first UASB was denitrification using carbon from within the raw water, the A/O process served to initiate shortcut nitrification, and the function of the second UASB was to realize nitrogen removal. The system's final effluent presented COD, ammoniacal nitrogen, and TN levels of 70 mg/L, 11.3 mg/L, and 39 mg/L, respectively. The denitrification contribution rates by the three reactors were 24.6%, 49.6%, and 16.1%, respectively.

Phan et al. used a two-stage reactor to treat old leachate in which internal circulation ANAMMOX was implemented [56]. The influent's ammoniacal nitrogen and nitrite concentrations were 235–655 mg/L and 261–858 mg/L, respectively. Due to the excellent performance of the internal circulation system, the ammoniacal nitrogen volumetric loading rate exceeded 10 kg N-NH_4_^+^/m^3^·d. A high TN removal rate of 9.52 ± 1.11 kg N-NH_4_^+^/m^3^·d was observed when the TN concentration of the influent was 1500 mg/L. The specific ANAMMOX activity was found to be 0.598 ± 0.026 g N_2_-Ng/VSS·d. DNA analysis showed that *Candidatus Kuenenia stuttgartiensis* was the dominant species in the reactor at 37.45%.

### 6.3. Summary of TN Removal by Activated Sludge Processes

TN removal has been a problem in all previous research and activities associated with leachate treatment. As new technologies, ED and ANAMMOX have positive and negative characteristics. The biggest advantage of ED is that no external carbon source is needed to obtain high TN removal and operation is simple. A disadvantage of this process, however, is that it can only process the leachate when it contains enough carbon and this limits its application.

ANAMMOX is a hot water treatment technology, and its advantages are low costs, high TN removal rates, and not needing an external carbon source. However, the drawbacks of ANAMMOX are that it is a complicated process and management of the system is difficult. Moreover, ANAMMOX bacteria are difficult to obtain and slow to grow. The domestication of the system is problematic.

## 7. Summary

In summary, due to low costs and good results, activated sludge processes are the preferred technology for landfill leachate treatment. Discharge that meets the required standards would be easy to realize if the problems of organic matter and TN could be solved. TN removal from leachate is particularly difficult; conventional disposal processes are currently low in efficiency or high in cost, and this makes it difficult to apply them practically to leachate treatment. New treatment processes, such as ED and ANAMMOX have significant advantages. The design of these processes and their parameters should be pursued and optimized to aid the water treatment industry; future research should explore and focus on these core leachate processes.

## Figures and Tables

**Table 1:** Characteristics of landfill leachate with different periods.

Leachate type	Early	Medium-term	Old
Landfill useful life (years)	<5	5–10	>10
pH (−)	6.5–7.5 (7.0)	7.0–8.0 (7.5)	7.5–8.5 (8)
COD (g/L)	10–30 (15)	3–10 (5)	<3 (2)
BOD/COD (−)	0.5–0.7 (0.6)	0.3–0.5 (0.4)	<0.3 (0.2)
NH_4_^+^-N (mg/L)	500–1000 (700)	800–2000 (1000)	1000–3000 (2000)
COD/NH_4_^+^-N	5–10 (6)	3-4 (3)	<3 (1.5)

^∗^The values in parentheses are typical values.

**Table 2:** The organic treatment efficiency of landfill leachate by the anaerobic activated sludge process.

Processes	COD of leachate	Removal rate	Reference
UASB	5400 mg/L–20000 mg/l	80%	[16]
UASB	8550 mg/L–12500 mg/L	80%	[17]
UASB	12350 mg/L–47800 mg/L	80%	[18]
UASB	3500 mg/L–4200 mg/L	76%	[19]
Anaerobic MBR	2800 mg/L–5000 mg/L	95%	[20]
Anaerobic MBR	13000 mg/L	62%	[21]
EGSB	33000 mg/L	85%–90%	[22]

**Table 3:** The organic treatment efficiency of landfill leachate by aerobic activated sludge process.

Processes	COD of leachate	Removal rate	Reference
SBR	1348 mg/l	COD 80%–85% BOD 99%	[23]
SBR	1040 mg/L–4870 mg/L	COD 70%	[24]
MBR	2200 mg/L	COD 60%	[25]
MBR	5445 mg/L	COD 98.1%–99.25%	[26]
AO	2000 mg/L–3000 mg/L	COD 40%	[17]
